# Utilizing Social Media Advertisements and Participant Social Networks to Recruit African American Breast Cancer Survivors: Design and Rationale

**DOI:** 10.3389/fpubh.2022.931102

**Published:** 2022-07-11

**Authors:** Avonne E. Connor, Kate E. Dibble, Kala Visvanathan

**Affiliations:** ^1^Department of Epidemiology, Johns Hopkins Bloomberg School of Public Health, Baltimore, MD, United States; ^2^Department of Oncology, Johns Hopkins Sidney Kimmel Comprehensive Cancer Center, Baltimore, MD, United States

**Keywords:** study protocol, social media advertising effectiveness, African American (AA), breast cancer, cancer survivors, social networks (online), recruitment

## Abstract

**Purpose:**

Our objective is to pilot an advertisement-driven sampling procedure among African American (AA) breast cancer survivors living in Maryland. These pilot study methods will inform a future population-based study of AA breast cancer survivors at high risk of poor outcomes due to biological differences and social inequities.

**Methods:**

This cross-sectional study utilizes an innovative, social media-based advertisement campaign with an associated social media study page to recruit 100 AA breast cancer survivors. Participants are biologically female, aged 18 and older, identify as AA/Black, have a diagnosis of breast cancer, and reside in Maryland. A preset “Audience” was created *via* Meta (formerly Facebook) to automatically target potential interest in the online study *via* geolocation and public social media interests (estimated range = 101,000 women). Eligible participants complete an online survey including demographic and clinical characteristics, cancer screening, healthcare access, and utilization, COVID-19 impact, quality of doctor-patient communication, and preferences for future study participation.

**Results:**

Recruitment began on 5 January 2022 and remains ongoing. As of 7 June 2002: 124 completed the screener, 110/124 (88.7%) consented passively, 24/110 (21.8%) started but did not complete survey, 86/110 (78.1%) completed the survey.

**Conclusions:**

Results from this study will inform a statewide multilevel prospective population-based study to improve health behaviors, disease management, and self-efficacy of chronic disease management among AA breast cancer survivors.

## Introduction

African American (AA) breast cancer patients have the highest breast cancer mortality rates and shortest overall survival than any racial/ethnic group of women in the US (1). The cause of the disparities in breast cancer and overall survival among AA women is multifactorial and reflects the interplay between biological factors (differences in tumor biology, advanced stage of disease,) and socioeconomic disparities. Co-existing comorbidities have been shown to increase risk of breast cancer mortality by 20–50% and competing-cause mortality up to 6-fold (2). Unequal access to opportunities and resources such as wealth, income, and education can also significantly influence access to high-quality healthcare services, such as disease prevention and screening, early detection, and treatment services (1).

AA breast cancer patients remain *underrepresented* in biomedical research and are considered “*hard-to reach*” for study participation (3, 4). Historically, recruitment and retention of minority cancer survivors into epidemiological studies has been challenging (5). There are barriers (i.e., physician notification and approval required might slow study recruitment, low response rates, lengthy recruitment process, distrust of research studies) associated with using state cancer registries to recruit cancer survivors to population-based studies (6). Therefore, to develop generalizable studies to reduce cancer health disparities, it is imperative to identify novel strategies for the recruitment (and retention) of AA cancer survivors.

Meta (formerly Facebook), and now Meta-acquired Instagram, have been increasingly utilized for research recruitment purposes (7–9). According to national data, approximately 7-in-10 Americans use social media to connect with others, with young AA women making up approximately 40–70% of Meta and Instagram users (10). Within the cancer realm, both platforms have been used to recruit for small qualitative studies to large-scale online randomized clinical trials, finding that most recruitment practices have been increasingly positive (11–16), especially since these websites have introduced “Audiences,” allowing advertisers to identify groups of individuals based upon age, sex, and location. While the use of social media including Facebook and Twitter has been found to be successful for recruiting young AA breast cancer survivors for study participation (17), this advertisement (or “ads”) feature has also shown to be beneficial for the recruitment of other hard-to-reach and specialized populations (8, 18–20).

The goal of this research is to conduct a pilot study of AA breast cancer survivors using social media recruitment strategies and to pilot study methods to inform a future population-based intervention study of AA breast cancer survivors at high risk of poor outcomes due to biological differences (comorbid conditions) and social inequities. The information gained from this research will enable us to implement a targeted approach to identify the most at-risk breast cancer survivors in Maryland for intensive lifestyle interventions. The current project will recruit this hard-to-reach population using social media (Meta and Instagram and associated targeted ads and study page) and sharing *via* social media profiles.

## Materials and Methods

### Study Design

A total of 100 AA breast cancer survivors (18 years of age and older) living in Maryland and diagnosed with breast cancer are being recruited from social media platform click-oriented ads *via* Meta and Instagram (estimated reach available for screening from Meta and Instagram combined = 101,000 individuals living in Maryland based on geolocation, interested [*via* social media activity] in breast cancer awareness, treatment, survivorship, support). All participants must identify as AA/Black and have been diagnosed with breast cancer (stages *in situ*-IV) and reside in Maryland (identified by geolocation).

Social media accounts are not being collected but are being utilized for ad purposes, as there is only direct contact between participant (or potential participant) and research team through Facebook study “Pages” direct messaging and *via* institutional email addresses. By private messaging through the “Pages” forum, research administration can view names and information (depending on participants' Meta and Instagram privacy settings) without research administration using their own social media accounts. Rather, they are directly conversing with participants *via* a “Page” front. Meta and Instagram are owned by the same parent company, making recruitment through ads straightforward. Meta allows two types of ad budgets: (1) Meta and Instagram's daily budget and (2) cost per thousand clicks (CPM). For the purposes of the current study, we utilized a daily budget, allowing $5–8 per day for each Meta and Instagram running for approximately 2 to 6 months or until recruitment ceiling is reached. For more details about the study advertisement, please see Supplementary Figure 1. Screenshot of Meta Ad. This study was approved by the Johns Hopkins Bloomberg School of Public Health Institutional Review Board.

### Study Procedures

Meta and Instagram have the capability to identify potential "Audiences” for ads based upon age, sex, geolocation, and interest in special groups (e.g., breast cancer treatment, awareness, support, survivorship). By creating this preset audience, Meta and Instagram displays our research post ad to individuals who live in Maryland on a smartphone, computer, or tablet while individuals are using Meta and/or Instagram. Responses will be limited to singular IP addresses to prevent multiple responses and reCAPTCHA was enabled to prevent internet bots [computer programs that operate as an agent for a user to simulate human activity (e.g., to complete online surveys falsely for compensation purposes)] and spammers (completing many surveys in a short period of time to “phish” for compensation). Advertisements will prompt participants to contact study personnel for the screener survey link by clicking the advertisement link. Clicking the advertisement link will reroute participants to the Meta study Page, where potential participants can directly message study administration. The study Page contains the following information for potential participants to view to ensure the legitimacy of the study: one post was pinned to the Facebook study page in September 2021 to describe the study eligibility criteria, study details, and how to participate; headshot photos of the study team (principal investigator and co-investigators); captions for each photo. Participants are also given the option to email the study administration directly (*via* JHU email) from information on the Meta study Page.

To prevent fraudulent use of the survey link by bots and spammers, we have avoided publicly posting the survey link on Meta (only posted in private Messages to participants) and periodically search for the RedCAP© link itself to see if it has been published elsewhere. We include a sentence in the passive consent about not compensating bots or spammers and use a screening mechanism to determine participant eligibility and use the reCAPTCHA function through RedCAP©. Within the survey, we have removed the use of a “back” button at the end of the questionnaire to prevent a resubmission of the survey. We also have added a timestamp at the beginning and end of the questionnaire through the RedCAP© platform to evaluate the length of time it takes participants to complete the survey. If the survey is completed within only a few minutes, we then determine if the data provided by the participant is valid.

**Figure 1:**
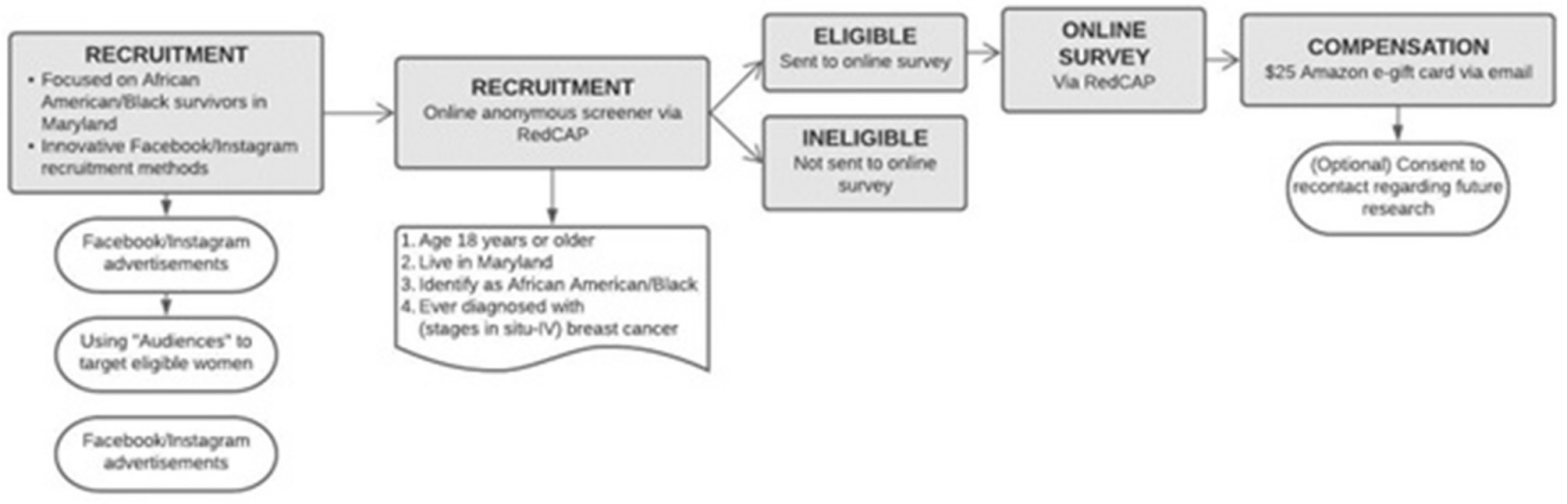
Study Procedures.

Evaluating eligibility according to recruitment standards (18+ years of age living in Maryland; identifying as AA/Black and have a diagnosis of breast cancer) takes place using an online, anonymous eligibility screening mechanism *via* RedCAP© managed by the Johns Hopkins University Information Technology department. Individuals who meet inclusion criteria for the current study are then redirected to the electronic consent page to review passive consent information and by clicking the “next” button they provide consent to participate in the full online survey. If the participant is not eligible, they are thanked for their interest but are informed that they do not qualify for the current study. Eligible participants will provide their email address for compensation and recontact purposes (regarding future contact for new studies). Upon completion of the online survey, participants are emailed a $25 Amazon e-gift card (as noted on the Meta ad) to the email address they have provided (see Figure 1 for Study Procedures).

### Online Survey

Online survey sections focus on several independent and dependent factors, including: demographic characteristics (current age, state of residence, county of residence, annual income, education, and employment), clinical characteristics (cancer diagnoses, stage of cancer at diagnosis, cancer treatments, recurrences, and metastases), recent and former cancer screening history [mammography, magnetic resonance imaging (MRI)], genetic testing information (if they completed genetic testing), healthcare access and utilization, health promotion questions (weight history, smoking, alcohol consumption, diet/nutrition, physical activity), COVID-19 impact, and quality of PCP-patient communication (treatment decisions, sufficient time, understanding, respectful, genuine, available treatment options, etc.). The online survey also collects preferences for future interventions, willingness to participate in medical record abstraction, and email addresses for compensation and future contact. Table 1 includes specific details of the Online Survey sections.

**Table 1:** Variables collected through online survey of AA/ Black breast cancer survivors.

***DEMOGRAPHIC AND CLINICAL***	***MEASUREMENT(S)***
Sociodemographic information	Current age, education level, ethnicity, race, marital status, sexual orientation, employment status, county residence, annual household income
Clinical information	Age at first breast cancer diagnosis, stage, treatments and side effects, treatment status (current, completed), recurrence, metastasis, relevant cancer screenings
Comorbid conditions (self)	NHANES Medical Conditions Questionnaire (MCQ) Charlson Comorbidity Index PROMIS Self-Efficacy for Managing Chronic Conditions – Symptomology PROMIS Self-Efficacy for Managing Chronic Conditions – Managing Medications and Treatments
Family history of breast cancer	Family history of breast cancer Genetic testing status
Body mass index	NHANES Weight and Height Questionnaire
Physical activity	NHANES Physical Activity Questionnaire (PAQ)
Smoking	NHANES Smoking – Cigarette Use Questionnaire (SMQ)
Physical environment and safety	US Determinants of Exercise in the US Women's Health Survey
Eating habits	NHANES Diet Behavior and Nutrition Questionnaire
Perceived health	NHANES Healthcare Utilization Questionnaire (HUQ)
Healthcare utilization	NHANES Healthcare Utilization Questionnaire (HUQ)
Healthcare discrimination	Everyday Discrimination Scale *via* Discrimination within Medical Settings
Healthcare access	Health Outreach Partners – Transportation and Health Access Ability to obtain medical care when needed Ability to obtain specialist care when needed Using emergency room for usual source of care Regularly visiting the doctor Distance traveled for care
COVID-19	Impact on participant lifestyle from Pandemic Stress Index (PSI) by Harkness (2020)
Preferences for future interventions	Preference for receiving intervention, frequency of intervention, preferred mode of reminders and frequency of reminders Hypothetical medical record abstraction question Email for payment

*NHANES (National Health and Nutrition Examination Survey)*.

### Statistical Analysis Plan

Responses from the online survey will be downloaded from RedCap© into SAS version 9.4 and Stata version 16. Prior to all analyses, all relevant study variables will be inspected through descriptive statistics and graphical techniques to assess distributional assumptions, identify outlying observations, and note the outcome trajectories. Missingness plots will be evaluated to determine the observed and missing data structures. Quantitative outcome variables will be transformed to approximate normality, if required. Baseline demographic variables for dropout and completed cases will be compared using chi-square tests for categorical variables and *t*-tests or Mann-Whitney tests for continuous variables.

The data collected through this pilot questionnaire and results from exploratory research aims will be used for preliminary data purposes for future grant applications. Descriptive characteristics (demographic, clinical, and lifestyle factors) will be analyzed in terms of frequencies, means, standard deviations using chi-square tests for categorical variables and *t*-tests or Mann-Whitney tests for continuous variables. To ensure that study participants recruited from this method are representative of the underlying population of breast cancer patients diagnosed in Maryland, we will compare the demographic and clinical characteristics to Maryland Cancer Registry data utilized for previous studies (21, 22). Due to the nature of this study (cross-sectional), crude and adjusted odds ratios and 95% confidence intervals will also be calculated between independent variables and dependent variables. Effect modification by socioeconomic status will also be explored.

## Preliminary Results

### Study Participants

Recruitment began on 5 January 2022 and remains ongoing. As of 7 June 2022: 124 have completed the screener, 110/124 (88.7%) have consented passively, 24/110 (21.8%) started but did not complete survey, and 86/110 (78.1%) have completed the survey. Table 2 includes descriptive characteristics of the study participants who have completed the survey as of 7 June 2022 (*n* = 86). The average ages at time of survey and at primary breast cancer diagnosis are 58.7 years (SD = 10.5; range: 32–81) and 48.5 years (SD = 10.5; range: 26–78), respectively. Most women live in Prince George's County (31.4%), Baltimore City (16.3%) and Baltimore County (15.1%), Maryland. For sociodemographic factors, 30.2% have a college degree as their highest level of education and 47.1% have an annual household income of $40,000–99,999 (Table 2).

**Table 2:** Preliminary descriptive results of study participants with completed surveys (*N* = 86).

**Characteristics**	***N* (%)**
***County of residence***	
Allegany	1 (1.2)
Anne Arundel	5 (5.8)
Baltimore City	14 (16.3)
Baltimore County	13 (15.1)
Calvert	1 (1.2)
Caroline	1 (1.2)
Carroll	1 (1.2)
Cecil	0 (0.0)
Charles	5 (5.8)
Dorchester	0 (0.0)
Frederick	3 (3.5)
Garrett	0 (0.0)
Harford	2 (2.3)
Howard	5 (5.8)
Kent	0 (0.0)
Montgomery	5 (5.8)
Prince George's	27 (31.4)
Queen Anne's	1 (1.2)
St. Mary's	0 (0.0)
Somerset	0 (0.0)
Talbot	0 (0.0)
Washington	0 (0.0)
Missing	0 (0.0)
***Annual household income***	
< $20,000	7 (8.2)
$20,000–$39,999	14 (16.5)
$40,000–$74,999	21 (24.7)
$75,000–$99,999	19 (22.4)
$100,000–$149,999	12 (14.1)
$150,000–$199,999	9 (10.6)
$200,000 or more	3 (3.5)
Missing	1 (1.2)
***Education***	
Less than high school	0 (0.0)
High school graduate or GED	11 (12.8)
Some college or technical/vocational school	18 (20.9)
College graduate	26 (30.2)
Some graduate school	7 (8.1)
Master's degree	18 (20.9)
Professional degree (JD, MD, etc.)	5 (5.8)
Doctoral degree	1 (1.2)
Missing	0 (0.0)
***Current marital status***	
Married or living as married	33 (38.8)
Divorced	21 (24.7)
Separated	3 (3.5)
Widowed	4 (4.7)
Single (never married)	24 (28.2)
Missing	1 (1.2)
***Current employment status***	
Working full-time	40 (46.5)
Working part-time	2 (2.3)
Full-time homemaker or family caregiver	2 (2.3)
Retired	27 (31.4)
Student	0 (0.0)
Unemployed	6 (7.0)
Other	9 (10.5)
Missing	0 (0.0)
***Living arrangement***	
Home owned or being bought (e.g., mortgage)	53 (61.6)
Rented	29 (33.7)
Other arrangement	4 (4.7)
Missing	0 (0.0)
***Health insurance at time of cancer diagnosis***	
No health insurance	3 (3.5)
Private insurance	57 (66.3)
Medicare	11 (12.8)
Medicaid	2 (2.3)
Cobra	1 (1.2)
Veteran's Affairs (VA)	2 (2.3)
Other government insurance	10 (11.6)
Missing	0 (0.0)
***Current health insurance***	
No health insurance	0 (0.0)
Private insurance	41 (47.7)
Medicare	27 (31.4)
Medicaid	5 (5.8)
Cobra	0 (0.0)
Veteran's Affairs (VA)	3 (3.5)
Other government insurance	10 (11.6)
Missing	0 (0.0)
***Type of primary breast cancer diagnosis***	
*in situ*	25 (29.8)
Invasive (stages I-IV)	59 (70.2)
Missing	2 (2.3)
***Metastases in primary breast cancer diagnosis***	
No	73 (84.9)
Yes	13 (15.1)
Missing	0 (0.0)
***Current treatment status***	
Currently undergoing treatment	21 (25.6)
Completed treatment	58 (70.7)
Did not receive treatment	3 (3.7)
Missing	4 (4.7)
***Second cancer diagnoses post-primary***	
No	74 (87.1)
Yes	11 (12.9)
Missing	1 (1.2)
***Other lifetime cancer diagnoses***	
No	73 (84.9)
Yes	13 (15.1)
Missing	0 (0.0)
***Breast cancer recurrence***	
No	70 (82.4)
Yes	15 (17.6)
Missing	1 (1.2)
***Consent to being recontacted within 5 years***	
No	1 (1.2)
Yes	85 (98.8)
Missing	0 (0.0)
***Hypothetical consent for medical record abstraction***	
No	14 (16.7)
Yes	70 (83.3)
Missing	2 (2.3)
	***M*** **(*****SD*****)**
Age at survey completion	58.7 (10.5)
Age at primary breast cancer diagnosis	48.5 (10.5)

### Ad and Page Statistics

**Figure 2:**
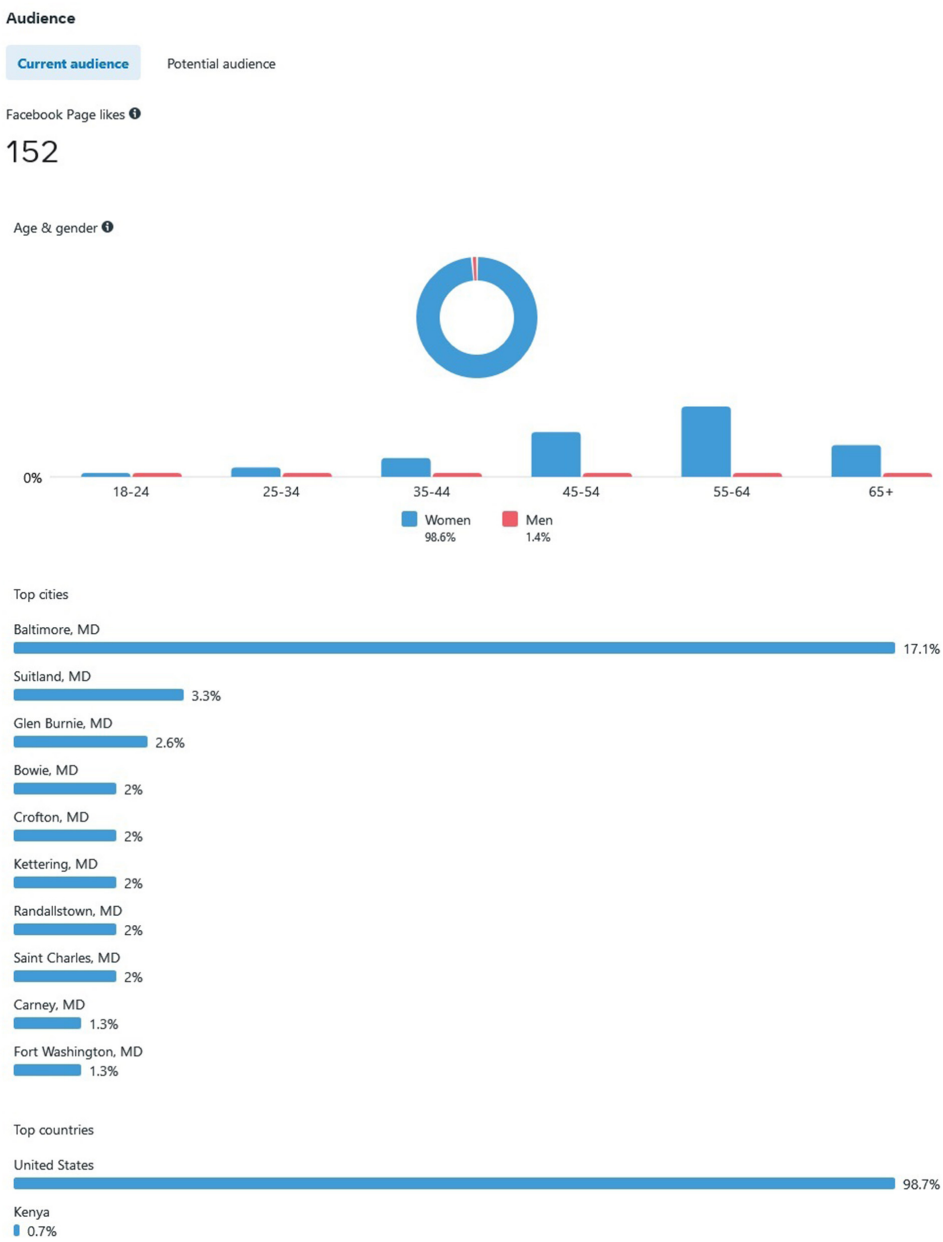
Meta Ad Insights from Eligible Target Population from 5 January 2022 to 7 June 2022.

Advertising through Meta is advantageous, as they provide ad-based statistics on how well the ad is performing. As of 7 June 2022, our Meta ad has reached 92,670 individuals, all residing in the state of Maryland. Most individuals encountered our ad on the Meta mobile app News Feed (*n*= 82,638, 89.1%) and videos feed (*n* = 14,032, 15.1%). Others viewed our ad *via* Meta Stories, mobile web News Feed, desktop News Feed, in-stream video, Marketplace mobile, Facebook search results, Instagram Stories, and Instagram Feed. By demographic, most individuals were female and 45 years and older. Out of the 92,670 that have viewed the ad, there were 1,991 (2.14%) generated link clicks of interest at $0.37 cost per click, 464 post reactions, 150 post shares, and 56 post comments. Figure 2 depicts the *projected* Audience for our ad, ranging from current age, Maryland county (the majority from Baltimore = 17.1%), and country (US) where the *target* Audience resides by IP address.

**Figure 3:**
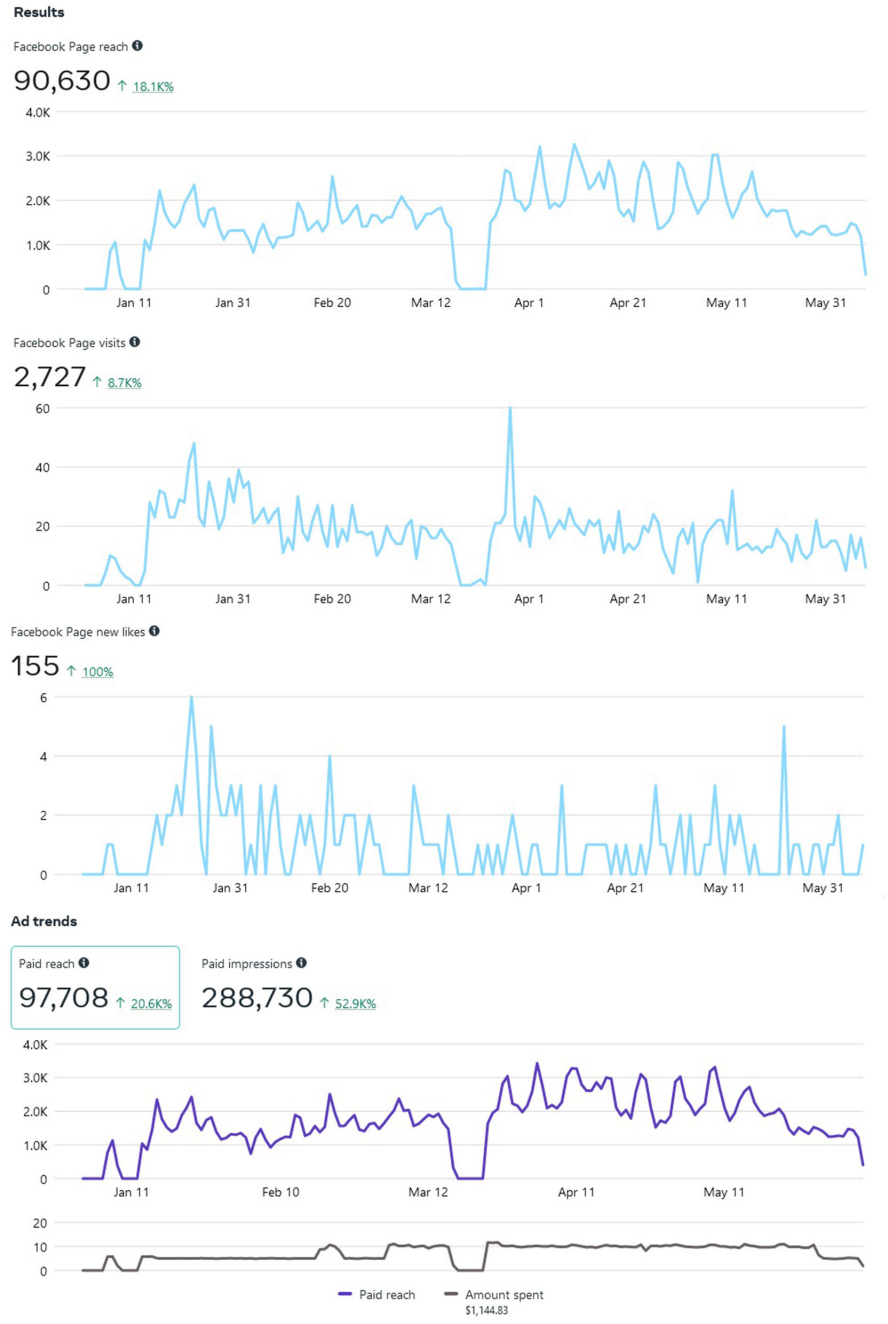
Facebook Page Reach, Page Visits, and Page Likes from 5 January 2022 to 7 June 2022.

Meta allows “Insights” into how well your Page is performing vs. how well it is being advertised. For example, our target Audience mostly consisted of those aged 25 to 65+. Our Page reached an estimated 90,630 individuals since inception on 5 January 2022 (note: this statistic is different than ad reach), and separately reached approximately 92,670 individuals *via* paid ads. Our study Page generated 2,727 distinct Page views and 155 Page likes as well (see Figure 3). Only one post was pinned to the Facebook study Page in September 2021, and as of 7 June 2022, 1,073 individuals have been reached (e.g., number of unique users who viewed the post) with 84 separate engagements, 62 likes, comments, and shares (e.g., study recruitment does not allow outside sharing due to spam/bots). Recruitment will remain ongoing until our recruitment ceiling is met (*N* = 100 completed interviews).

## Discussion

Our pilot study is examining the effectiveness of a new recruitment strategy that could increase the enrollment and retention of minority breast cancer survivors into community-based studies. To identify the most at-risk breast cancer survivors in Maryland due to comorbidities and social inequities, we will examine factors associated with health status (characterized by diagnosed comorbidities) and capture their self-efficacy for managing chronic conditions among this high-risk population. Maryland is the ideal state to conduct such a study given that the AA population represents more than 30% of the total population, unlike the US where the AA population is close to 14% (23). Maryland's population also has varying levels of economic position, as Prince George's County has one of the highest median household incomes in the US according to 2019 data ($84,920 compared to Baltimore City $50,379) (24). Therefore, Maryland is positioned as a significant resource to study breast cancer disparities.

There are benefits to using social networks and social media for study recruitment approaches. Study-associated Meta and Instagram “Pages” provide participants with direct and private messaging with study personnel, allowing for online assistance if needed to answer questions about the study or study updates (responses are provided within 24 h). The Meta study Page also connects survivors and family/friends of survivors with information to learn more about the study, breast cancer disparities, and the study investigators/team. Recent research has shown that cancer patients are now utilizing social media more during COVID-19 as a resource to answer questions related to their cancer experience (25, 26). We have found that breast cancer survivors recruited through Meta share the study information and questionnaire directly with their social networks on social media and with individuals who might not have Meta accounts. This form of friend-referral has shown to increase the rate of recruitment and interest in the study.

There are also limitations to consider with this type of recruitment and online study design. There are ethical issues relating to social media recruitment that have been noted in recent literature, most notably the respect for persons and their privacy as well as investigator transparency through truthfulness and honesty (27). While RedCAP© enables the reCAPTCHA as a form of protections to circumvent the online intrusion of bots and spammers, online studies have encountered this type of interference across social media platforms (28). Past literature has also noted spammers and bots in online social networking sites (29, 30), which have increased drastically in popularity during the COVID-19 pandemic (31). Due to the recency of issues such as these and the increase of online studies utilizing social media ads for recruitment, spammers and bots have become more of a persistent problem.

One recent study has suggested recommendations for ensuring data integrity with online studies that are susceptible to bot interference, including the use of strategies such as qualitative questions, duplicate demographic questions, and incentive raffles to reduce likelihood of mischievous respondents (32). For the current study, we initially had the survey link posted to the study Page and immediately encountered spammers. We then employed several mechanisms to deter bot interference and have established post-survey assessments to identify invalid cases as noted in the Methods. To date, we have not encountered any additional issues with bot interference or spammers.

## Conclusion

Although there exist limitations relating to social media recruitment, if done preemptively from study conception, securities can be put in place to prevent bots and spammers, a major issue for studies using this form of recruitment. Additionally, our study has found that the use of the study Page to filter fraudulent responses for study participation is a novel method to connect potential study participants to more information about the study and to interact with other interested social media users. Past literature on the use of social media among breast cancer survivors has shown that limited research has focused on non-Caucasian populations (33). Our study will add to the growing body of literature focused on the use of social media and networks for recruiting and conducting population-based studies among AA/Black breast cancer survivors. Initial study results will be disseminated through public talks/seminars to the local breast cancer survivor support groups located in Maryland and will be posted in aggregate form to the study Page. In collaboration with breast cancer focused-community organizations, results from this research will inform a statewide multilevel prospective population-based study to improve health behaviors, disease management, and self-efficacy of chronic disease management among AA breast cancer survivors at high risk of poor outcomes due to biological differences and socioeconomic disparities.

## Ethics Statement

The studies involving human participants were reviewed and approved by Johns Hopkins Bloomberg School of Public Health Institutional Review Board (IRB). Written informed consent for participation was not required for this study in accordance with the national legislation and the institutional requirements.

## Author Contributions

AC conceived the study. AC and KD designed the recruitment strategy and analyzed the data. AC, KD, and KV contributed to the writing of the manuscript. All authors contributed to the article and approved the submitted version.

## Funding

This work was supported by the American Cancer Society (MRSG-19-010-01-CPHPS). KD was supported by the National Cancer Institute (T32CA009314).

## Conflict of Interest

The authors declare that the research was conducted in the absence of any commercial or financial relationships that could be construed as a potential conflict of interest.

## Publisher's Note

All claims expressed in this article are solely those of the authors and do not necessarily represent those of their affiliated organizations, or those of the publisher, the editors and the reviewers. Any product that may be evaluated in this article, or claim that may be made by its manufacturer, is not guaranteed or endorsed by the publisher.

## Supplementary Material

Supplementary Figure 1 Screenshot of Meta Ad. This study was approved by the Johns Hopkins Bloomberg School of Public Health Institutional Review Board.
