# Supplementary figures and images for: Utilizing Social Media Advertisements and Participant Social Networks to Recruit African American Breast Cancer Survivors: Design and Rationale
Source: Front Public Health. 2022 Jul 11;10:931102. doi: 10.3389/fpubh.2022.931102 (PMC9309894; doi:10.3389/fpubh.2022.931102)

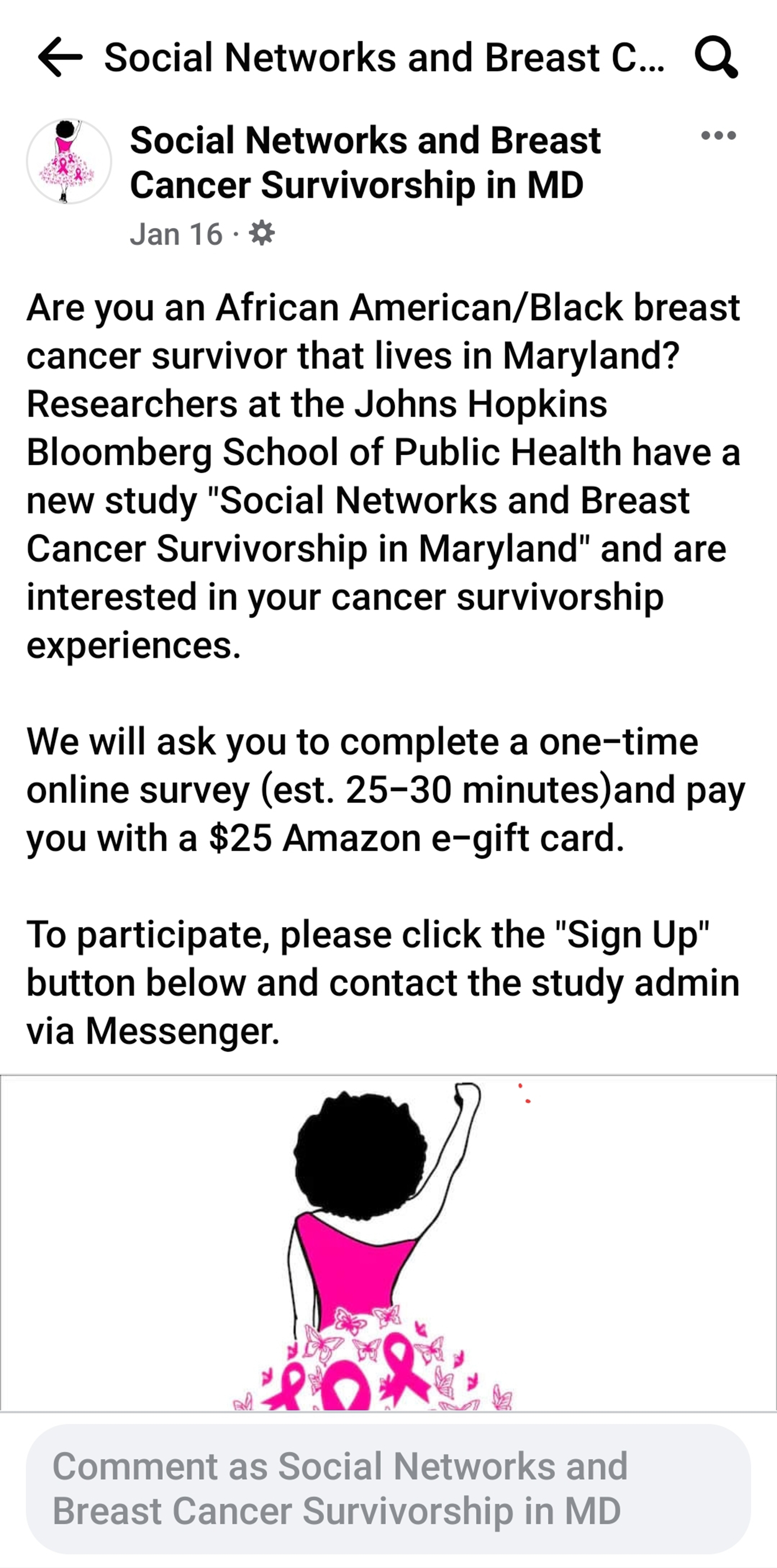

Supplement: Supplementary Figure 1 — Screenshot of Meta Ad. This study was approved by the Johns Hopkins Bloomberg School of Public Health Institutional Review Board. [file Image_1.JPEG]
